# “We are stewards and caretakers of the land, not exploiters of resources”: A qualitative study exploring Canadian farmers’ perceptions of environmental sustainability in agriculture

**DOI:** 10.1371/journal.pone.0290114

**Published:** 2023-08-15

**Authors:** Jocelyn Carmichael, Abbey Cran, Felicia Hrvatin, June Matthews

**Affiliations:** School of Food and Nutritional Sciences, Brescia University College at Western University, London, Ontario, Canada; Florida Atlantic University Charles E Schmidt College of Medicine, UNITED STATES

## Abstract

Environmental sustainability in agriculture is a key component of discussions to address the current climate crisis; unfortunately, many people (including researchers) presume that only certain types of agriculture (e.g., organic, local) are environmentally sustainable. Non-farmers also fail to acknowledge that many farm practices, including grazing animals, mitigate climate change. Farmers’ perceptions about environmental sustainability are important because their livelihoods, and those of future generations, depend on their commitment to sustainable environmental practices. The purpose of this qualitative research was to understand Canadian farmers’ perceptions of environmental sustainability, how they are implementing strategies that contribute to sustainable food production, and the challenges they face. Fifty-two farmers, representing 48 farms and over 1000 years of farming experience, participated in comprehensive in-depth interviews. Four farms were in British Columbia; 13 in the Prairies; 26 in Central Canada; and five in Eastern Canada. A wide variety of farm types (e.g., fruit/vegetables, livestock, grains) and sizes (2 to 6500 acres) were included in the study. Farmers’ perceptions of environmental sustainability coalesced into four main themes: (1) definitions of sustainability and environmental sustainability, (2) current practices, (3) farming as an identity, and (4) challenges. Many participants explained that they already use sustainable practices and technology, contrary to prevailing opinion that entire food systems need to be transformed to be sustainable. As new agricultural policies and educational curricula are developed, information provided to students, policy makers, and the public must be accurate, balanced, evidence-based, and respectfully consider all perspectives, especially those of farmers.

## Introduction

As the need to mitigate climate change becomes more urgent, sustainability has become a popular topic. Some researchers have assigned three pillars to sustainability: environmental, social, and economic [1]. Sustainability, as it relates to agriculture, can be defined as “a holistic, long-term approach to business on-farm, that means maximizing economic and environmental stability, equity, and health of the farm, business and family” [2]. Almost half (45%) of Canadians define sustainable food as having a “positive impact on climate change and the environment” [3, p.20]. The Brundtland Report noted that the exact dimensions of sustainability depend on economic, social, and ecological conditions of each country, and there is no one-size-fits-all policy or practice [4].

Similarly, there is no single definition for sustainable agricultural practices (SAP) [5], but they generally include practices that conserve soil and water quality, protect biodiversity, and efficiently manage pests. The specifics of these practices, as well as motivations for using them, depend on farmers themselves, what they grow or raise, and land characteristics [2,6]. Census data, for example, can provide information on agricultural practices; however, they do not tell us why farmers are adopting these practices.

Many researchers have examined farmers’ motivations and barriers to SAP. Ranjan et al. [7] synthesized findings from 49 qualitative research studies that examined farmers’ adoption of conservation practices and programs in the United States of America. Most studies revealed economic factors as a motivator, given that some practices are costly to implement, and monetary incentives are necessary to make adoption feasible. Other motivators included government cost-share programs, the integration of crop production with raising livestock, and farmers’ stewardship identity [7]. Barriers to adoption of SAP included physical incompatibility (e.g., lack of land availability), increased time and effort, or desire to maintain status quo [7]. Qualitative research exploring Canadian farmers’ and consumers’ perceptions of beneficial agricultural management practices showed that farmers “did not think of these as environmentally sustainable practices so much as the way they farm. They do these things because it is better for the sustainability (environmental, social, and financial) of their business” [8, p.3]. Canadian consumers expressed difficulty in providing an assessment of Canadian farming practices because of lack of awareness [8]. As one participant explained, “I don’t know what they are doing, so I can’t say if they are doing enough. I think they are doing their best with the resources they have” [8, p.16]. While there is increased demand from many sectors for farmers to adopt SAP to address climate change and meet Sustainable Development Goals, many people are unaware of the obstacles that farmers face and what they are already doing to enhance environmental sustainability on their farms.

Barriers to the adoption of technical innovations for sustainable development in the agricultural sector can be described as external (e.g., lack of policies or their unfavourableness) or internal (e.g., inertia and resistance to unfamiliarity) [9]; however, it may be challenging to address specific barriers if there is not agreement on the classification of the innovation in question (i.e., product, process, organizational, or marketing). For example, Campuzano et al. [9] classified organic agriculture as a process innovation. Some would argue that the organic industry is primarily a marketing innovation. Campuzano et al. [9] also classified Genetic Engineering (GE) as a product innovation [9]; however, GE is not a product; it is a process.

It is also problematic when only certain food production techniques (e.g., organic), and/or the scale at which foods are produced and sold (e.g., local), are deemed sustainable. For example, Thompson et al. [10] investigated the adoption intensity of SAP in Europe. They categorized farms into conventional, a mix of conventional and alternatives, or only alternative practices. Conventional farms were those that only used chemical products (not approved by organic regulations); mixed farms used chemical products and alternatives such as biological controls; and alternative farms only used “sustainable practices” [p.4]. In addition to incorrectly assuming that only those farms following organic regulations were using SAP, they reverse coded the use of conventional pesticides and herbicides “so that farms which did not use these products were given additional credit” [p.4]. These authors not only founded their research on incorrect assumptions, but they also suggested their results will have “important implications for sustainable agricultural policy development” [p.1]. The consequences are that an ill-informed public [11] and environmental non-governmental organizations are unduly interfering with agricultural policy and advocating regulatory barriers for innovative agricultural technologies [12], particularly if they are reading this type of peer-reviewed article.

Farmers are experts in their field and knowledgeable about sustainable land practices; however, their voices are not often heard [13]. Fortunately, this is changing, as more qualitative research studies are being published. In addition to the studies synthesized by Ranjan et al. [7], researchers have explored farmers’ perspectives in China, to understand their adoption of precision agriculture to deal with environmental challenges [14]; in England, to determine the best methods for delivering pollution mitigation advice [15]; in Senegal, to understand farmers’ perceptions of maintaining healthy dairy cattle to meet increasing demand for milk [16]; in Ireland, to explore dairy farmers’ values related to grass-based production practices [17]; in Germany, to understand farmers’ adoption of beneficial soil microbe solutions [18]; and in the United States of America, to explore the potential for increased adoption of conservation tillage [19]. These research articles are not only expanding our understanding of SAP beyond the data captured in national census reports, but they are also giving farmers’ voices a wider reach, particularly through open access publications. Recent research from Canada has tapped into farmers’ knowledge and perceptions about mental health, primarily through a large survey, with an additional 54 qualitative interviews with farmers [20].

Despite this increase in knowledge transfer, most non-farmers do not understand the complexity of agriculture, and the challenges farmers face. Ninety-one percent of Canadians claimed they knew “little or nothing about modern farming practices” [21, p.8]. With almost 75% of the Canadian population living in an urban center [22], it is not surprising that many Canadians are increasingly disconnected from their food system and unsure if it is headed in the right direction [23]. With the rise of social media and celebrity influencers, consumers are overwhelmed with information and opinions, and uncertain where to find credible information.

In relation to the sustainability of food production, Canadians are concerned about climate change and the rising cost of food [24]. The Canadian government is also concerned about the future of agriculture in relation to climate change and is committed to investing in sustainable agriculture while responding to the needs of farmers; environmental sustainability is one of five pillars of reform in the Canadian Agricultural Policy Framework for the 21st Century [25,26]. The Agri-Communication Initiative aims to connect consumers to their food and highlight the steps producers are taking to use sustainable practices [27]. The Canadian government has also been developing The Green Agricultural Plan, which aims to enhance resilience to climate change, as well as biodiversity protection and water and soil health [28]; however, the proposal to reduce emissions from fertilizer 30 percent by 2030 is encountering significant resistance from farmers [29].

As new policies like these are developed, it is important to learn about farmers’ perceptions, not only because they are experts, but also because they are ultimately in control of the practices they use [13]. Perception can be defined as “the way an individual observes, understands, interprets, and evaluates” an action, experience, or policy [30]. Farmers’ knowledge of their land, developed from years of experience in a local environment, influences their perception of changes in their environment and associated risks [13]. In turn, their assessment of changes and risks compared to benefits determines their support for conservation or environmental initiatives [30]. Quantitative research on agricultural practices can lack depth on the complex systems surrounding farmers and the plethora of factors that influence their decisions; thus, qualitative research can increase our understanding of farmers’ motivations and challenges [13]. Furthermore, to ensure that policies are not driven by misinformed public opinion, both consumers and decision-makers must understand farmers’ perceptions and knowledge so that the policies created are ones that farmers can and will adopt [31,32].

It is also important to research sustainability in Canada’s food systems as the topic gains popularity in education systems. For example, Ontario’s new elementary science curriculum explores the topics of food systems and climate change [33]; however, it lacks appropriate context and teaching materials. Sustainability has also recently been emphasized in the Canadian Integrated Competencies for Dietetic Education and Practice Performance Indicators [34]. These Performance Indicators influence what will be taught in Canadian university food and nutrition programs to students, who, in their future careers as food and nutrition experts, will have the opportunity to educate patients and clients about food systems and sustainable choices [35,36]. Thus, it is imperative that the information taught is accurate, balanced, evidence-based, and respectfully considers all perspectives, including those of farmers.

The purpose of this study was to understand Canadian farmers’ definitions and perceptions of sustainability, how they are implementing strategies to achieve sustainable food production on their farms, and the challenges they face in doing so. While interview questions addressed environmental, social, and economic aspects of sustainability, this paper will focus on the environmental aspect to allow sufficient opportunity for their voices and own words to be heard. The economic and social components will be reported separately.

## Materials and methods

In this descriptive qualitative study [37], the researchers assumed a constructivist approach [38] and explored, through in-depth conversations with participants, their perspectives of the situation being studied [37]. Descriptive qualitative research is a well-accepted, rigorous, and useful approach for graduate students when basic description and summary of a phenomenon is desired [37]. To be included in the study, participants had to produce food through farming on land, own or rent the land on which they farm, and reside in Canada. English-speaking farmers were recruited through Canadian agriculture-related organizations, an advertisement in a rural magazine, and snowball sampling to participate in a telephone/video interview between January and March 2019. Most early participants were from [blinded province for review]; therefore, recruitment was extended until March 2020 to achieve maximum variation sampling [39], which, for this study, included diverse geographic locations and types of farms.

The Letter of Information–including the risks and benefits of participating in the study; assurances that participants could withdraw at any time and/or not answer all questions; as well as details on data security, confidentiality, and storage–was read to each participant at the beginning of their online/telephone interview. Participants then provided informed verbal consent, which was documented by the interviewer. A semi-structured interview guide (S1 File), developed by the researchers based on a review of the literature and expert opinion, included the following broad research questions: (i) What comes to mind when I say the word sustainability? (ii) What does food production sustainability mean to you? and (iii) How would you describe environmental sustainability as it relates to farming? Generic prompts (e.g., Can you tell me more about that? What do you mean by…?) encouraged participants to expand on their responses. The interviewers also periodically summarized participants’ responses and asked for confirmation or clarification during the interviews (i.e., member checking) to enhance trustworthiness and decrease researcher bias [40]. At the end of the interview, participants were asked several demographic questions (S2 File), the answers to which were documented on hard copy. A $25 honorarium was offered to each participant in appreciation for their time and effort. This study, including the procedure for obtaining and documenting oral consent, was approved by the Non-Medical Research Ethics Board at [blinded for review] University, Approval #112911.

All interviews (except one) were audio-recorded, transcribed verbatim by the graduate student researchers and undergraduate research assistants, independently coded by the researchers, and analyzed concurrently throughout data collection using the constant comparative method [41]. Initial themes were organized in Microsoft Excel and updated or expanded with sub-themes as additional data were collected and analyzed. A post-graduate student from the Diploma in Dietetic Education and Practical Training program at [blinded for review] was also invited to independently conduct secondary analysis of the transcripts. Analyst triangulation [42] allowed multiple ways to understand the data, minimized bias and selective perception, and contributed to a comprehensive analysis (i.e., credibility/internal validity) [40]. Trustworthiness was also enhanced by memo writing to document emerging interpretations and decisions made throughout the research process (i.e., dependability/reliability), and by in-depth descriptions of both the phenomenon (farmers’ perceptions of environmental sustainability) and the participants, which can help readers determine applicability of the findings in their own context (i.e., transferability/external validity) [40]. The student researchers and their faculty advisor met weekly during this iterative and reflective analytic process to de-brief and come to thematic consensus (i.e., confirmability/objectivity). Credibility was also enhanced by peer debriefing [43] as the student researchers discussed the research process, themes, and context amongst themselves to enhance their interpretation of the data.

The researchers also engaged in reflexivity on how their backgrounds and knowledge of the phenomenon (or lack thereof) influenced both processes and outcomes [44]. The student researchers were not farmers but were familiar with current issues in Canadian agriculture through participating in an undergraduate agriculture course (taught by the faculty advisor). The faculty advisor is a Registered Dietitian and Professional Home Economist with a farming background and many years of experience in conducting qualitative research studies.

Themes and sub-themes that were identified from transcript data are supported by representative quotations from one or more of the participants to add trustworthiness and transparency to the findings [45]. Including participants’ own voices deepens readers’ understanding of participants’ perspectives and experiences, reinforces the researchers’ interpretations, and shows the richness of the data [45]. To protect confidentiality, each participant was assigned a random number between 1 and 52, and, consistent with established guidelines for reporting qualitative research [46], quotations have been labelled with a unique identifier (e.g., ON26F = [Ontario], Participant #26, Female).

## Results

Fifty-two farmers across Canada were interviewed online via Zoom (Zoom Video Communications Inc., 2019) or by telephone. There were 48 interviews in total (four interviews had two participants each). Years of farming ranged from two to 50, representing, in total, over 1000 years of farming experience. Participant characteristics can be found in Table 1.

**Table 1:** Participant characteristics (n = 52).

Characteristic	% (n)
**Age (y)**	
18–29	15 (8)
30–39	25 (13)
40–49	23 (12)
50–59	17 (9)
60–69	13 (7)
70+	6 (3)
**Sex**	
Male	65 (34)
Female	35 (18)
**Education**	
High school	4 (2)
College/Apprenticeship	40 (21)
University (undergraduate)	42 (22)
University (post-graduate)	14 (7)
**Marital Status**	
Married	75 (39)
Not married	25 (13)
**Off-farm work** ^a^	
Full-time	29 (15)
Part-time	27 (14)
None	44 (23)

^a^ Interviewees only, not spouses/partners.

Compared to recent census data, where more than half (60.5%) of Canadian farmers are 55 years of age or older [47], only 20% (10/52) of our participants were older than 60 years of age. The percentage of participants who identified as female (35%; 18/52) reflects the percentage of female farm operators in Canada (30.4%) [48]. The education level of participants who had college/apprenticeship (40%; 21/52) was comparable to 2016 Census of Agriculture data (35%) [49]; however, the percentage of participants (56%; 29/52) who had university degrees (undergraduate and post-graduate) was much higher than the 2016 Census of Agriculture (18%) [49]. Finally, 56% (29/52) of participants engaged in off-farm work, a higher percentage than the percentage of Canadian farmers reporting off-farm work (47.7%) [47].

Of the 48 farms represented in the study, four were in Canada’s most western province (British Columbia [BC]); 13 were in the Prairie provinces (Alberta [AB], Saskatchewan [SK], and Manitoba [MB]); 26 were in Central Canada (Ontario [ON]); and five were in Eastern Canada (Nova Scotia [NS] and New Brunswick [NB]). Farms were categorized by size according to the number of acres owned and rented (which represents each farm’s total land area being farmed/pastured): small (less than 99 acres), medium (100–999 acres), and large (greater than 1000 acres). All farms in British Columbia were small (mean: 18 acres; range: 2–45), with no acres rented. Participants in the Prairie provinces had mostly large farms (mean: 3312 acres; range: 120–6500); 10 (77%) of them had rented acreage (mean: 1031 acres; range: 60–2000). Most farms in Ontario were medium in size (mean: 221 acres; range: 20–450). Thirteen of these farms included an average of 132 rented acres (range: 1–400). The five farms in Eastern Canada (NS, NB) were a mix of small, medium, and large.

Participants with small farms (n = 11) produced vegetables, fruit (apples, wild blueberries), and hops, as well as cut flowers and shrubs/trees. A few raised animals (laying hens, pigs, mixed livestock, cow-calf). Medium-sized farms (n = 22) primarily had dairy cows, beef cattle, broiler chickens, laying hens, and sheep. These farms also produced grains, forages, and legumes. Some farms in this category produced fruits (apples, strawberries) and vegetables (field-grown and greenhouse). Large farms (n = 15) produced a variety of crops (canola, wheat, corn, soybeans, barley, yellow peas, quinoa, flax, oats, and mustard), as well as forages/cover crops (alfalfa, timothy, rye grass). A few also had laying hens, broiler chickens, dairy cows, and beef cattle.

### Themes

Participants’ perceptions of environmental sustainability coalesced into four main themes and associated sub-themes (Table 2). Theme 1 captured the dimensions of participants’ definitions of sustainability in general and environmental sustainability in particular. Participants revealed their dedication to continuous improvements by enumerating many of their current practices; thus, Theme 2 consists of an extensive list of SAP employed by these participants. Throughout the interviews, it became clear that farming was more than just an occupation, making farming as an identity the basis for Theme 3. In Theme 4, participants revealed the challenges they faced in their quest to achieve, or receive credit for, sustainable environmental practices on their farms. Differing views were also documented.

**Table 2:** Themes and sub-themes developed through analysis of qualitative interviews.

Main Themes	Sub-themes
1. Definitions of sustainability and environmental sustainability	Minimizing impacts and waste Enhancing biodiversity Preserving land for future generations
2. Current practices	Improvement and adaptation Improving fuel efficiency Consultation with experts
3. Farming as an identity	Stewards of the land Feelings of pride
4. Challenges	Economic challenges Public misperception of agriculture Lack of government support Climate change

### 1. Definitions of sustainability and environmental sustainability

Many participants found it challenging to define sustainability in general. A descriptive analogy was provided by a farmer from Eastern Canada: *“It’s like a milk stool I use*, *you know*, *it’s a tripod*. *You need all three pillars [environmental*, *economic*, *social]”* (NS45F). A farmer from Central Canada expanded on this: *“If you don’t have all three legs*, *it’s going to fall over”* (ON30F). Five other participants echoed this sentiment, recognizing that long-term sustainability requires each ‘leg’ to be strong.

When defining environmental sustainability, the overall goal was to *“to do the best we can to protect the resources around us to the best of our abilities”* (ON39F). Another aspect was to aim for balance, as expressed by this grain farmer from Manitoba: *“Everything that we do has to be balanced so that you are replenishing*, *whether it’s seed*, *fertilizer*, *the soil*, *the moisture”* (MB17M). Three sub-themes were also identified: minimizing impacts and waste, protecting biodiversity, and preserving land for future generations.

#### Minimizing impacts and waste

Participants defined environmental sustainability as limiting their impact on the environment by, for example, maintaining a balance between inputs and outputs, or operating within a “closed-loop” system, as described by one beef farmer: *“not really relying on many other inputs from other sources [outside of the farm]”* (ON12M). An apple grower explained that environmental sustainability means *“minimizing the impact of my practices on the environment… minimizing sprays*, *minimizing disturbance to the soil”* (ON15M). A specific dimension of minimizing impacts was minimizing waste. Farmers described the controls used to minimize their waste and outputs from livestock, watering, and other operations. A sheep farmer explained, *“We’re always keenly aware of how we feed our animals*, *and what happens to their manure and their urine”* (ON26F). Greenhouse operators also talked about minimizing waste, as explained by this participant: *“Environmental sustainability definitely comes down to controlling our outputs–wastewater*, *waste leaves*, *and waste crop–so they do not affect the environment”* (ON31M).

#### Enhancing biodiversity

Participants expressed appreciation for the contributions of all organisms on their farms, from the cows that provide nutrient-rich manure, to the lady bugs that combat other pests. As a dairy farmer explained, *“We want a productive and healthy ladybug population because they attack a primary bug that eats and destroys crops*. *So*, *we wanna make sure that certain insects and wildlife are protected”* (ON25M). Participants also highlighted their land as a unique and rich ecosystem that was important to enhance and protect. As a small beef farmer explained,

*Cattle are more important than people think*. *Their manure is like black gold*. *There’s a gazillion grassland birds that need pastures and hay fields to live*, *and they come with the cattle and eat the bugs and everybody’s happy* (ON10F).

#### Preserving land for future generations

Participants also emphasized the importance of family. The desire to maintain the productivity of family farms provided a strong rationale for participants’ efforts regarding environmental sustainability. Indeed, seventeen participants (33%) defined environmental sustainability as preserving their land for future generations. A participant from a large prairie farm summed it up in the following way:

*I’m in it for my children*. *I’m not in this to make millions and get out*. *I’m in it for the next generation*. *I want to see my children do better than I did and grow food for the world*. *I want to leave my farm in better shape for my son*, *my daughter*, *or my nieces than when I got it* (SK42M).

### 2. Current practices

All participants mentioned specific techniques they were using to improve their farm’s environmental sustainability. These included practices related to soil, water, energy, and biodiversity (Table 3). A large grain and oilseed farmer stated, *“On our operation*, *we do no tillage*. *We’re trying to build our organic matter [in the soil]”* (SK36M). Many farmers used multiple techniques in combination, or one technique that accomplished multiple goals. For example, a fruit and vegetable grower said, *“the idea of cover crops is that it adds organic matter to your soil*, *stops erosion in the winter*, *and in the spring when it’s still wet*, *it keeps your soil down more*.*”* (ON07M). Similar to pest and weed control practice of large grain growers, a hop farmer explained, *“We do weekly scout walks to monitor pests and*, *ideally*, *through that monitoring*, *we can minimize our sprays”* (BC43M).

**Table 3:** Sustainable agricultural practices employed by Canadian farmers to achieve environmentally sustainable food production.

Resource	Sustainable Agricultural Practice
Soil	Cover crops to prevent erosion (alfalfa, red clover, hay)
	Windbreaks
	Crop rotation
	No-till
	Rotational grazing
	Ruminants (farm animals)
	Nutrient management plans
	Fertilization
	“One pass” system to add nutrients
	Perennial crops
	Permanent pastures
	Soil sampling
Water	Riparian strips/grass buffers
	Barriers to prevent livestock access
	Filtration systems
	Closed-loop circuits
	Cover crops to prevent runoff
	Drip irrigation
	Water tests
Energy	Solar panels
	Efficient livestock/poultry housing
	More energy-efficient machinery
	No-till
	Lower wattage lights and fans in farm buildings
Biodiversity	Appropriately timed pesticide application
	Adherence to pesticide/herbicide/insecticide regulations
	Cultivation of wildflowers or leaving areas ‘wild’
	Preservation of habitats for birds
	Integrated pest management programs

#### Improvement and adaptation

Current practice improvements and adaptations emerged as a key sub-theme under current practices, with one farmer’s comment reflecting the view of several participants: *“I feel this is probably this is one of our greatest successes; the fact that we’ve adapted*, *we’ve become more efficient”* (SK42M). Participants were proud of their proactive stance to learn about new technologies and implement them on their farms. A grain, oilseed, and pulse farmer from the prairies expressed this prevalent sentiment by saying, *“Land is a resource that needs to be maintained and retained and improved continually to do what we do*. *So*, *we’re pretty early adopters of whatever technologies that are developed to help us in those regards”* (MB32M). Reasons for improving practices included maximizing production while limiting costs and impacts on the land. Improved techniques were credited with accomplishing multiple goals at once, as explained by a beef cow/calf operator who also grows corn, soy, and wheat: *“We are trying to use the most advanced technology we can to maximize our crop production and make a living doing it…As we move along*, *our technology is allowing us to adapt and become better stewards of the land”* (ON04M).

#### Improving fuel efficiency

Participants from across Canada mentioned that new techniques and equipment were ways to increase energy efficiency. One participant, with a large farm in a prairie province, said, *“We’re using less fossil fuels because of technology as well*. *We’re leaving less of a carbon footprint”* (AB16F). Similarly, on another large prairie farm: *“We farm probably triple*, *if not quadruple the amount of land that we did 25 years ago and we’re probably using the same amount of fossil fuels”* (SK42M). Further, another farmer in Eastern Canada explained, *“For no-till*, *when you’re making fewer passes*, *you’re saving on fuel”* (NS45F).

Some farmers noted they had begun using solar power or were reducing their hydroelectricity use with updated, efficient lights and equipment in farm buildings. A farmer who raises laying hens stated, *“We produce enough solar energy to cover our needs for almost a full year”* (MB48M).

#### Consultation with experts

Farmers also described the importance of seeking improvement to expand their knowledge and learn new techniques. This included attending conferences or consulting experts: *“We get the advice of soil scientists to better indicate to us what we should be placing on that land to make sure that we’re making environmentally conscious decisions”* (SK42M). Others worked closely with local authorities. For example, a cow/calf farmer said, “*We worked with our local Agricultural Environmental Officer through Ministry of the Environment”* (ON39F). Another, whose farm abuts a conservation area and river, said, *“We work through the Upper Thames River Conservation Authority”* (ON03M).

### 3. Farming as an identity

Throughout the interviews, farming was described not only as an occupation, but also as an identity with environmental overtones.

#### Stewards of the land

Almost half of the participants described themselves as *“stewards and caretakers of the land”*, which was expanded by a beef farmer who added, *“and not exploiters of resources”* (ON21M). One large grain farmer’s response aptly reflected this theme: *“I think by and large farmers by default are good environmentally*, *they’re good stewards of the land*. *You know we’ve definitely reduced erosion*. *We’re more efficient with using technology”* (AB34M). Many participants believed that all farmers are good stewards of their land because it is part of being a farmer, in that *“A farmer’s livelihood depends on their stewardship of the land”* (ON04M). This was a main responsibility of farming, not only to keep the farm economically viable, but also because it was important *“to do the right thing”* (MB48M). The conviction that they were doing what was best for the greater or common good in terms of environmental sustainability, and not simply making decisions for their own benefit, provided a solid foundation for their identity, and was clearly linked to the research topic, as a market gardener and beef farmer explained, *“We are the stewards of the land*. *And stewardship and sustainability go hand in hand”* (ON19M). Another beef farmer expanded on this by saying, *“Farmers are doing the right things*. *They’re doing things more sustainability all the time*, *learning new technologies and ways of learning things*, *and how to do things more efficiently”* (ON21M).

#### Feelings of pride

All participants exhibited pride in their work, and they balanced the health of their land with the need to be productive. Farmers indicated they were proud of the standards on their farms, the quality of their products, and their role as stewards. A farmer raising broiler chickens explained, *“We eat the food that we produce*, *and we do a great job of producing food”* (ON24M). A dairy farmer said, *“The product that we are growing is the best quality it can be*, *whether we are feeding the cows or whether we’re feeding humans”* (ON25M). A vegetable grower also differentiated between Canadian practices and global practices: *“I think here in Canada anyway we do a really*, *really good job”* (ON22M), demonstrating pride in Canada’s regulations and standards of practice. A greenhouse operator added, “*We have our own [standards] that go above and beyond [mandatory standards and guidelines] as well”* (ON29M).

Another aspect of pride was having a farm operated by the same family for multiple generations. Three-quarters of the participants talked about this generational aspect of farming. One farmer, operating a large farm in Eastern Canada, said their son was the tenth generation on their farm, with *“one plot of land continuously farmed [by their family] since the 1700s”* (NS45F).

### 4. Challenges

For the last theme, all participants acknowledged that farming is a challenging profession. This included the sub-themes of economic challenges, public opinion, lack of government support, and climate change.

#### Economic challenges

Many noted that farming is challenging and stressful, primarily because it is a high-risk profession. Volatility in markets, range in profits, and the threat of extreme weather events or pests damaging crops often result in a huge loss of income. A greenhouse operator explained,

*If it’s in the winter and it’s cold out and your heating system shuts down*, *you could lose the crop*. *If it’s in the summer and it’s hot out and your irrigation system stops working*, *you could lose the crop*. *If you get a virus*, *you can lose the crop*. *If you get a pest that is deadly and spreads very quickly*, *you can lose the crop*. (ON31M).

Almost all participants described economic constraints as the largest challenge when considering environmental sustainability. Although farmers want to do what is best for their land, it is difficult to invest in new measures because, as a vegetable grower explained, *“It costs money*. *And you don’t get an immediate return*. *So*, *if you’re not a second or third generation farmer*, *you have to make big payments*. *Sometimes you have to weigh your options*, *right*?*”* (ON22M). Additionally, interventions such as riparian strips may necessitate the conversion of profitable land into unusable land. Several participants mentioned that if there was more support or funding from the government, they would be able to implement more environmentally sustainable measures.

#### Public misperception of agriculture

Outside of economics, the biggest challenge was public misperception of agriculture. Many participants expressed concern that the public is disconnected from farming, both in terms of location and personal networks. As a grain farmer explained, “*I think there’s a disconnect*, *especially the newer generation because they’ve lost any connection [with agriculture] they may have had*. (AB34M)

Farmers reported that although most Canadians do not farm, they still have strong opinions about how farmers should operate. Many participants reported feeling frustrated that they were taking care of their land, but still vilified by the public: *“We’re all doing a great job with [increasing sustainability]*. *We just have to get the general public to understand that*, *so they don’t impose ridiculous regulation on us”* (ON04M). Confusion compounded their feelings of frustration, as expressed by a dairy farmer:

*If you spray a pesticide*, *you eliminate the pest*, *and you save that whole crop*. *Of course*, *if the regulatory or the consumer environment doesn’t like that idea*, *not because it isn’t safe–it’s been proven to be–but if the feeling is they don’t like it*, *then what do you do*? (ON25M)

#### Lack of government support

Participants commented that misinformed government regulations can hinder farming practices. One participant from the prairies described a date, set by their provincial government, for when they can apply synthetic fertilizer or manure each year, regardless of the weather or field conditions. Regulations were reported as challenges, not benefits, and contributed to feeling a lack of support, as voiced by this sheep farmer: *“We seriously need real support*, *and we need [the government] to understand what we’re doing”* (ON26F). An oilseed farmer from the prairies explained how this additional support was directly linked to environmental sustainability: *“They encourage us to use proper equipment to work the land*, *but the government isn’t throwing enough money back at the farmers to go get those extra pieces of equipment that would provide those opportunities”* (AB16F).

#### Climate change

Climate change and severe weather events were also reported as challenges. A farmer with laying hens explained, *“All our erosion control structures and grassed waterways are designed for a 1-in-50-year weather event*. *Well*, *sometimes [climate change] pushes it*, *as we’re getting more severe weather”* (ON03M). Additionally, some participants discussed unique challenges to interventions, such as the difficulty implementing no-till in thick clay soil, or experiencing cooler soil due to no-till, which impeded germination.

#### Differing views

While most farmers had similar answers to the research questions, there was some variation in opinions. It was noted that not every farm operation was engaged in best practices. One farmer, for example, said, “*It is a huge concern to us when we see huge operations abusing the land*, *not having enough land for their manure and waste products*, *but that’s just the nature of [any profession]”* (ON20F). Two participants, one from a prairie province in Western Canada and the other from a farm in Central Canada, had opposing views on the Verified Beef Production Plus Program:

*In my opinion*, *McDonalds is using the word ‘sustainability’ because it’s a catch phrase these days*. *They require you to be a member of a verified beef program*, *for example*, *where you have to give your cattle certain shots*, *vaccinations*, *and that kind of thing and we don’t do that* (AB14F).

*I just today signed up for the Verified Beef Plus Program which allows you to use that logo so that producers and feedlots who are looking for those calves where the cow-calf producer has followed you know due diligence*, *codes of ethics*, *and sustainable practices* (ON10F).

Similarly, some farmers expressed frustration with organic farming, especially when they felt it was falsely advertised as better than conventional farming; others were passionate organic farmers. An organic grain farmer explained that the *“best way to increase environmental sustainability*, *increase carbon levels in your ground*, *and the whole biodiversity is to have cattle or other ruminants on your farm”* (ON18M). Although he admitted that the cattle industry is getting a bad reputation, he confidently stated that, *“If it’s done properly*, *it can be the best way to improve environmental sustainability”* (ON18M). Many farmers said that choosing a technique or system is a trade-off and every approach has pros and cons. A corn/soy/wheat/bean farmer explained, *“Without no-till farming*, *we’re going to have more erosion*, *and [to do no-till] we need herbicides to control the weeds”* (ON23M). Another corn/soy/wheat/beef farmer said,

*There is pros and cons to both organic and conventional food production*. *The one downfall [to organic] is that there is the mechanical cultivation*. *You do have to cultivate*, *and you do have to plough*, *which burns more fossil fuels* (ON12M).

## Discussion

Most farmers are experts in managing their land and adapting to changes; however, they are not often given a platform to explain their practices [13] and their voices are drowned out by non-farmers who have ample time to express their opinions and ideologies on social media. Consistent with previous research, participants had difficulty defining the abstract concept of sustainability [36,50,51]. Notably, participants found it easier to define environmental sustainability. Once the concept was linked to their profession, they were able to identify specific dimensions of it.

Participants’ definitions confirmed previous observations that environmental sustainability encapsulates more than reducing greenhouse gas emissions or eliminating pesticides; there are geographical and socio-economic nuances, as well as intricacies such as the beneficial role of ruminants in some ecosystems [52,53]. The farmers in this study showed an understanding of this and demonstrated a complex and holistic view of sustainability. While most farmers gave similar underlying definitions of sustainability, such as preserving their land for the future, there was variation in how they achieved it, demonstrating the need for flexibility within SAP and policies encouraging it.

Many participants noted that they already use sustainable practices and technology. While the majority of Canadian farmers are over 55 years of age [47], 80 percent of our participants were under 55. This may translate into greater interest and engagement in SAP, as well as a greater awareness of current public discourse surrounding sustainability. It has been shown that innovation in agricultural technology helps increase environmental sustainability [51,54]. This was confirmed by the farmers’ descriptions of increased efficiency and decreased impacts on their land due to investment in new equipment and knowledge. The younger age of participants and their higher level of education than the average farmer in Canada may explain this investment in new technology. All participants reported using at least one SAP, which matches Statistics Canada’s findings that an increasing majority of Canadian farmers use SAP [47]. Although consumers’ opinions of Canadian agriculture vary, most farmers, regardless of whether they are conventional or organic, are dedicated and committed to using safe, sustainable techniques [23,47].

Conserving energy and fuel was a common theme among participants. Statistics Canada reported a 66.5% increase in solar energy on farms in 2021 compared to the last census [47]. Although the farmers interviewed for this study may not represent the national farm population, several were successful in using solar energy on their farms. Energy sources may be an area of transition in future years; however, the success of this transition will be predicated on adequate financial support from the government.

Farming has a unique position as a profession, lifestyle, and identity [55,56]. The number of participants who self-identified as *“stewards of the land”* shows that Canadian farmers take pride in their farms, which creates meaning in their lives and motivates them to adopt sustainable measures in the interest of preserving their homes. Similarly, succession planning was a key motivator for pursuing environmental sustainability, and the ability to pass land on to future generations was used to define environmental sustainability. This complements previous findings that showed when a family member is a successor to a family farm, environmental measures and schemes are more favourable [57].

It has been well-documented that the cost of farming is a major source of stress for farmers, and a major barrier to the adoption of sustainable techniques [7,20,58,59]. This was also supported by recent stakeholder feedback from Canada’s Strengthened Climate Plan [28]. The frequency that cost was mentioned by participants in the current study indicates that it remains a major stressor for Canadian farmers. Farmers may be deterred from implementing environmental interventions if they are expensive and time-consuming. Tight profit margins limit the amount of capital farmers can invest in new technology and education, especially when there is a delayed return on investment or loss of revenue due to trial and error. This is particularly evident in a business such as farming, where the success or failure of a crop is not evident for at least one whole production year.

As indicated by participants in this study, and in line with previous research that suggested financial motivation is associated with farmers adopting new, more sustainable technology [7], one solution to this challenge would be financial incentives to support farmers’ adoption of sustainable/best management agricultural practices [59]. This would remove one of the largest barriers to SAP and encourage more farmers to adopt them [58]. Since these interviews were conducted, there have been some advancements towards increasing the accessibility of SAP in Canada. Notably, the Agricultural Clean Technology Program aims to provide farmers with funding to invest in new technologies and machines that will reduce greenhouse gas emissions and increase efficiency [26]. The federal government is also soliciting feedback on a proposed Sustainable Agriculture Strategy with the goal of continuing Canada’s capacity to support global food security while also improving the agriculture sector’s environmental performance [60]. Complementing the value of qualitative research, one of the guiding principles in its development will be “listening to the needs and concerns of farmers since they know their land best” [60]. It is hoped this farmer-focused approach will also yield a strategy that reflects farmers’ needs and capacities within the broader context of the economic and social challenges they experience.

Farmers in this study cited public opinion as a major challenge. Non-farming Canadians, especially those living in urban areas, are disconnected from Canada’s agriculture sector and lack agricultural literacy, a situation that has persisted for decades [61,62]. For example, participants in the current study were highly educated and reported professional development efforts such as attendance at conferences and collaboration with experts; however, one of the most common descriptions of farmers used by Canadians has been “simple” [23, p.7]. Non-farmers’ lack of knowledge about farmers and their work also contribute to conflicting views on agricultural practices. Although most Canadians think Canadian farmers are trustworthy [23,62], they are concerned about farmers’ treatment of livestock, genetically modified foods, pesticides, climate change, and sustainability [23,63]. There is also a tendency to favour a ‘naturalistic’ approach to farming, especially concerning animal welfare [63,64]; however, non-farmers’ perceptions of ‘natural’ approaches, such as organic or free-range, should not be conflated with actual, long-term sustainability of food production. Indeed, researchers have shown that the relatively low yields of organic farms–an estimated 30 percent lower than conventional farms [65]–may result in more land being used for agricultural production, with a loss of biodiversity greater than the on-farm benefit of organic practices [66].

Given the lack of agricultural literacy among non-farmers, their opinions and ideologies should not qualify as rationale for prohibiting certain agricultural practices [51,63]; however, they can and do influence policy development and impair farming technology innovations [11]. This is of particular concern for genetically modified (GM) crops. Over 4400 science-based risk assessments have documented the safety of GM crops, and economic assessments have quantified yield increases; however, there is still resistance to the adoption of GM crops in many countries, primarily through “political interference by environmental non-governmental organizations through campaigns of misinformation” [12, p. 602]. This has had a significant negative impact not only on global food security, but also on any attempt to achieve sustainable food production amidst a growing climate crisis. Participants in this study expressed their frustration with non-farmers’ misperceptions and their ability to influence farmers’ practices based on their opinions. Non-farming consumers must understand that farmers are already engaging in SAP, although it may not look like what they expect and may not be achievable at the rates they anticipate. There is always room for improvement, but farmers need to be recognized for the steps they are already taking [28].

Similarly, poorly informed government regulations impaired some participants’ operations. Canada’s recent plan to reduce synthetic fertilizer use [67] has received pushback for being unrealistic, and detrimental to profits and crop yields [68]. The farmers in this study emphasized that they do not feel supported by the current system; therefore, governments need to support farmers in what they are already doing and provide educational or financial support to facilitate further improvements. This is especially important as farmers’ incomes are at risk of being affected by climate change, and they bear the responsibility of adapting food systems to these changes [13]. A larger focus on providing accurate, balanced, and evidence-based agricultural education may also lead to greater public and government support for farmers.

Finally, as in any profession, participants held varying perspectives on topics relevant to their field. For example, some farmers promoted organic over conventional farming methods, yet all farmers and all farming methods are needed to provide food for a growing world population. Others differed in their support of the Verified Beef Production Plus Program [69], the goal of which is to ensure sound animal care, biosecurity, and environmental principles and practices. Both farmers and non-farmers should have a choice when it comes to the types of food products they wish to raise, produce, or eat, and the many ways in which they can do that sustainably. It is also important for non-farmers to understand that there are trade-offs between economic, environmental, and social sustainability. For example, many people may not realize that the more environmentally sustainable farmers are, the less likely they are to be economically sustainable [70]. Furthermore, changing from chemical crop protection to mechanical practices (oft-promoted solutions to achieve environmental sustainability) may adversely affect farmers’ working conditions and, in turn, the social sustainability of farms and rural communities [71].

Supply chain issues and empty shelves during the height of the Covid-19 pandemic reminded non-farmers that our food system is as complex as it is valuable, and improved public perception of agriculture and farmers [71]. In addition, for the first time, sustainability and environmental concerns ranked in the top five list of life concerns for Canadians [23]. There is also a better understanding that transporting food from farm to fork depends on the work and health of many individuals. Additionally, farming cannot be performed remotely, and during the pandemic, many farmers were further prevented from adapting to a “new normal” by poor internet access. Future research could examine farmers’ latest views on environmental challenges and new policies aimed at increasing the sustainability of agriculture. As these topics continue to change, research should be updated to accurately reflect farmers’ perceptions and challenges.

Farmers in this study were proud of their work and viewed their agricultural practices as already being environmentally sustainable, as evidenced by the fact that many were on farms which had been in their family for generations. Indeed, the list of SAP given by participants is indicative of a wide-ranging commitment to environmental sustainability for the benefit of future generations. Despite economic challenges and public misperceptions, farmers deeply valued this aspect of their work and integrated their perceptions of environmental sustainability into their personal identity as proud and responsible stewards of the land.

### Strengths and limitations

There are several strengths of this study. Maximum variation sampling [39] was achieved, with participants representing a variety of farmers in terms of age, sex, and type of farm, as well as different geographical regions of Canada. The percentage of female participants in our study (35%) was comparable to the percentage of female farm operators in Canada (30.4%) [48]. Diligent and extended recruitment efforts resulted in a respectable sample size for a qualitative research project. It is also important for researchers beyond agriculture to investigate farmers’ perceptions, and the four dietetic students involved in this study not only expanded their own knowledge of agricultural practices and challenges, but they also translated that knowledge into discussions with peers, family members, and patients or clients, and through formal presentations and publications that have the potential to enlighten others about the dimensions of sustainable food production from the perspective of people who actually produce food.

The authors also acknowledge several limitations. Most farmers were white and lived in Ontario. This is not surprising, as there are more farms and more farm operators in Ontario than in any other province [48]. No farmers in the territories, Newfoundland and Labrador, or PEI volunteered to be interviewed. The interviewers were graduate students and not farmers; therefore, more detailed data may have been gathered by a more experienced interviewer with a background in agriculture. For example, no information was gathered regarding the percent of total farm income generated on-farm. Information about off-farm income was solicited only at the end of the interview as one of the demographic questions. The authors acknowledge that if a farmer farms 5 acres and earns 90% of their total income off farm, then profitability and sustainability might be less of a concern. Thus, the perspectives of farmers who had very small farms may have been affected by the relative unimportance of farm income to total income. Self-selection bias was also a limitation, as only farmers who were willing to be interviewed participated in the study. Finally, data were collected immediately prior to the Covid-19 pandemic, and farmers’ views as well as their financial situations may have since changed.

This paper can inform multiple audiences, including students, teachers, members of the non-farming public, and policy makers, that many farmers are already engaged in sustainable food production, contrary to prevailing opinion that entire food systems need to be transformed to be sustainable. While agricultural practices can always improve, we must not dismiss certain food production approaches or assume that only one farming approach can solve all problems. All farmers should be valued for their hard work and their dedication to producing food for a growing world population, the majority of whom, by choice or by circumstance, are unaware of the complexity and realities of food production and the challenges that farmers face.

## Supporting information

S1 File Semi-structured interview guide. (DOCX)

S2 File Demographic questionnaire. (DOCX)
