# Supplementary material for: “We are stewards and caretakers of the land, not exploiters of resources”: A qualitative study exploring Canadian farmers’ perceptions of environmental sustainability in agriculture
Source: PLoS One. 2023 Aug 15;18(8):e0290114. doi: 10.1371/journal.pone.0290114 (PMC10427016; doi:10.1371/journal.pone.0290114)
Supplement: S1 File — (DOCX) [file pone.0290114.s001.docx]

**Semi-Structured Interview Guide**

**Project Title:** Exploring Farmers’ Perspectives of Food Production Sustainability

**Oral Interview Preamble**

Thank you for agreeing to participate in our study. The purpose of this study is to explore farmers’ perspectives of food production sustainability so that the viewpoints of those who produce food are included in how food production sustainability is defined and taught to students and the public.

University dietetic programs are expanding their curricula to include more instruction on food sustainability. As a Food and Nutrition student, I think it is important that the perspectives of those who produce food are included in how sustainability is defined and taught.

I will be audio-recording our conversation to ensure accuracy. You can skip any questions you may not wish to answer or ask me to turn off the recorder and end the interview at any time without any penalties. If you decide after the interview that you do not want part or all of your comments to be used, you can ask to have them withdrawn and the audio file destroyed.

I will be using the information that I collect from this interview, and several others, for my graduate research project. Every effort will be made to maintain strict confidentially over the information you provide. No one except the researchers will have access to your information. Some quotes from the interview may be used in the research report; however, your name will not appear in any reports of this study and any quotes used will not reveal your identity.

As we talk, I may ask you to explain or expand on what you say. There are no right or wrong answers. I want to understand *your* experiences, perspectives, and opinions.

Before we begin, I will read the Consent Form, and invite you to verbally indicate that you agree to participate in our study. At the end of our talk, you can decide if you are comfortable allowing me to use all or parts of our conversation in our research.

The interview will take 45 minutes to 1 hour. First, we will talk about sustainability in general and food production sustainability in particular. We will then discuss multiple aspects of food production sustainability that relate to your farm and your community. By food production we mean growing or raising food on land. Finally, we’d like to get your suggestions about effective ways to inform students and the public about the depth and breadth of the meaning of food production sustainability.

**Interview Questions:**

**Introduction**

1. First, what comes to mind when I say the word ‘sustainability’?
2. When we talk about ‘food production sustainability’ in particular, it can mean many things. What does that term mean to you? Keep in mind that we are talking about growing or raising food on land. *[Give participant time to answer, and probe minimally.]*

Sustainable food production can encompass 3 key aspects: environmental, economic, and social. Let’s start with the environmental aspect.

**Environmental**

1. Please tell me how you would describe environmental sustainability as it relates to farming.

*Probe*: What are some challenges in achieving long-term environmental sustainability of food production?

1. What suggestions do you have (e.g. who, what, when, where, why, and how) to teach students and the public about environmental sustainability of farming/farm communities?

Thank you very much. Let’s now talk about economic sustainability.

**Economic**

1. Please tell me what economic sustainability means to you as a farmer.

*Probe*: What are some challenges in achieving long-term economic sustainability of food production?

1. What suggestions do you have (e.g. who, what, when, where, why, and how) to teach students and the public about the economic sustainability of farming/farm communities?

Thank you, we will now discuss the third aspect, social sustainability.

**Social**

1. Please explain what it means for a farm to be socially sustainable within its community?

*Probe*: Do farmers in your community have access to education / health care / religious institutions / recreational facilities / community groups / mental health supports / etc.?

1. What suggestions do you have (e.g. who, what, when, where, why, and how) to teach students and the public about the social sustainability of farming/farm communities?

**Conclusion**

1. We have now talked about economic, environmental, and social sustainability related to food production. Is there one area that you think is the most important?

*Probe*: Can you explain why you feel this is the most important aspect?

1. Is there anything that we haven’t talked about today that you would like to share?

Thank you for your time. I’d like to briefly summarize what we’ve talked about today. Please tell me if I’ve understood you correctly. Could you please fill out this demographic questionnaire? Thanks again for participating in this study. Please choose a gift card you would like to receive electronically in appreciation for your time.
