# Supplementary material for: “We are stewards and caretakers of the land, not exploiters of resources”: A qualitative study exploring Canadian farmers’ perceptions of environmental sustainability in agriculture
Source: PLoS One. 2023 Aug 15;18(8):e0290114. doi: 10.1371/journal.pone.0290114 (PMC10427016; doi:10.1371/journal.pone.0290114)
Supplement: S2 File — (DOCX) [file pone.0290114.s002.docx]

**Demographic Questionnaire**

**The following questions will help us describe our research participants. Your answers will be aggregated with all other interviewees and will not be linked to your responses to the interview questions.**

1. What type of livestock and/or crops do you manage and/or grow?

_________________________________________________________________________________

1. Where is your farm located? _______________________________________ (area of province)
2. What is the size of your farm? ___________________ (acres)
   1. Approximately how many acres are owned? ______________
   2. Approximately how many acres are rented? ______________
3. How many years have you been farming? _________
4. Do you derive income from off-farm occupation(s)? _________ (yes/no)
   1. Approximately how many hours ______ or days ______ per week do you work off-farm?

**We would appreciate some additional information about you to help us better understand our findings.**

1. Highest level of education completed:

Public School __________

High School __________

College __________

Apprenticeship __________

Undergraduate __________

Graduate __________

Post-Graduate __________

1. Age: __________
2. Sex: __________
3. Marital status: __________________

10. Ethnicity/Ethnic background: ______________________
